# Spatiotemporal Patterns of Schistosomiasis-Related Deaths, Brazil, 2000*–*2011

**DOI:** 10.3201/eid2110.141438

**Published:** 2015-10

**Authors:** Francisco Rogerlândio Martins-Melo, Marta Cristhiany Cunha Pinheiro, Alberto Novaes Ramos, Carlos Henrique Alencar, Fernando Schemelzer de Moraes Bezerra, Jorg Heukelbach

**Affiliations:** Federal Institute of Education, Science and Technology of Ceará, Caucaia, Brazil (F.R. Martins-Melo);; Federal University of Ceará, Fortaleza, Brazil (F.R. Martins-Melo, M.C.C. Pinheiro, A.N. Ramos Jr, C.H. Alencar, F.S.M. Bezerra, J. Heukelbach);; James Cook University, Townsville, Queensland, Australia (J. Heukelbach)

**Keywords:** Schistosomiasis, spatial analysis, ecological study, epidemiology, mortality, vector-borne infections, Brazil, Schistosoma, trematodes, parasites

## Abstract

We analyzed spatiotemporal patterns of 8,756 schistosomiasis-related deaths in Brazil during 2000–2011 and identified high-risk clusters of deaths, mainly in highly schistosomiasis-endemic areas along the coast of Brazil’s Northeast Region. Schistosomiasis remains a neglected public health problem with a high number of deaths in disease-endemic and emerging focal areas.

Schistosomiasis is a neglected tropical disease (NTD) caused by infection with *Schistosoma* spp. trematodes and a public health problem worldwide, mainly in areas without access to safe drinking water and adequate sanitation (*1**,**2*). Brazil is the most heavily affected country in the Americas (*1*), with about 2.5 million–6 million infected persons (*3*) and 700–800 deaths are reported annually (*4*). The disease’s continued expansion because of human migration from schistosomiasis-endemic to -nonendemic areas means schistosomiasis is increasingly considered an emerging disease in Brazil (*5*). Using different spatial analytical approaches, we examined spatiotemporal patterns and determined high-risk clusters for schistosomiasis-related deaths in Brazil.

## The Study

We analyzed death certificate data obtained from the Brazilian Mortality Information System (http://tabnet.datasus.gov.br/cgi/sim/dados/cid10_indice.htm) and used the 5,565 municipalities of residence in Brazil as geographic units of analysis. We included deaths occurring during 2000–2011 for which schistosomiasis (code B65, International Classification of Diseases, Tenth Revision [ICD-10]) was recorded as underlying or associated (contributing) causes of death (multiple causes of death) (*6*). Deaths with unknown municipality of residence were excluded. Population data at the municipality level were obtained from the Brazilian Institute of Geography and Statistics (http://tabnet.datasus.gov.br/cgi/deftohtm.exe?ibge/cnv/popuf.def).

To minimize random variations, especially in municipalities with small populations and rare events, we calculated average annual death rates (per 100,000 inhabitants) at the municipality level over the entire period (average annual number of deaths/population size during the middle of the study period). We then calculated smoothed death rates by using the local empirical Bayes method (Technical Appendix). Presence of global and local spatial autocorrelation was evaluated by using Global Moran’s I and Local Moran’s I statistics (*7*), respectively (Technical Appendix). A retrospective space-time scan statistic (*8*) was used to identify statistically significant high-risk spatiotemporal clusters (Technical Appendix). Primary (i.e., most likely) and secondary clusters were detected by using the log-likelihood ratio test; clusters with maximum log-likelihood ratios were considered primary. 

**Figure 1:**
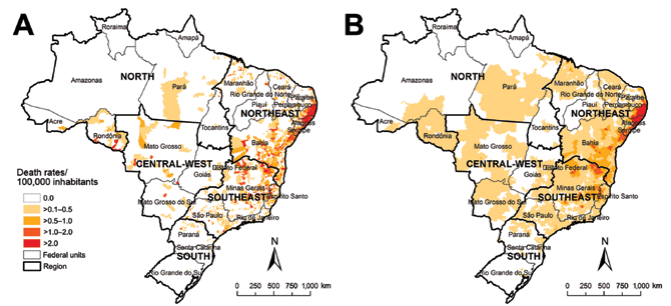
Spatial distribution of average annual crude (A) and Bayesian-smoothed (B) rates of schistosomiasis-related deaths, by municipality of residence, Brazil, 2000–2011. Empirical Bayesian smoothing estimates of rates of schistosomiasis-related deaths were performed by using TerraView software version 4.2 (Instituto Nacional de Pesquisas Espaciais, São Paulo, Brazil). Data were mapped by using ArcGIS software version 9.3 (Esri, Redlands, CA, USA). In 2010, Brazil was divided into 5 geographic regions (South, Southeast, Central-West, North, and Northeast), 27 Federal Units (26 states and 1 Federal District), and 5,565 municipalities.

A total of 12,491,280 deaths were recorded in Brazil for 2000–2011. Schistosomiasis was identified in 8,756 deaths (0.07%), as an underlying cause in 6,319 (72.2%) and as an associated cause in 2,437 (27.8%) deaths. The nationwide average annual crude rate of death atttibuted to schistosomiasis (for underlying and associated causes) was 0.39 deaths (95% CI 0.37–0.42) per 100,000 inhabitants. Of 5,565 municipalities, ≈1,225 (≈22%) recorded >1 schistosomiasis-related death. Spatial distribution of average annual crude and smoothed death rates at the municipal level showed a concentration of municipalities with higher death rates (>1.0 death/100,000 inhabitants) along the east coast of Brazil’s Northeast Region, extending to the states of Minas Gerais and Espírito Santo (Figure 1, panels A, B).

**Figure 2:**
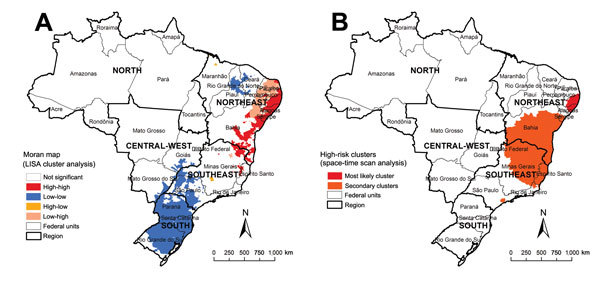
Spatial and spatiotemporal cluster analysis of rates of schistosomiasis-related deaths, by municipality of residence, Brazil, 2000–2011. A) LISA cluster analysis (Moran Map), based on Local Moran’s I index. B) Scan space-time clusters analysis, calculated by using Kulldorff’s scan statistics with SaTScan software version 9.1.1 (Harvard Medical School, Boston, MA, USA; Information Management Service, Silver Spring, MD, USA). Mapping and calculation of autocorrelation spatial analysis were conducted using ArcGIS software version 9.3 (Esri, Redlands, CA, USA). LISA, Local Index of Spatial Association.

Global Moran’s I index showed significant positive spatial autocorrelation (0.32, p<0.01). Local Moran’s I identified high-risk clusters (classified as “High/High”) of schistosomiasis-related deaths, corresponding mainly to municipalities with high rates shown in the descriptive maps (Figure 2, panel A). As with the concentration of high death rates, major high-risk clusters included a large geographic area on the east coast of the Northeast region (Figure 2, panel A).

Scan space-time analysis identified 3 spatiotemporal high-risk clusters (Figure 2, panel B; Table). Primary clusters were detected during 2001–2006 and represented 2,150 deaths in 191 municipalities distributed in 3 states in the Northeast region. The relative risk was 12.96 (p<0.01), and the annual crude rate was 4.0 deaths/100,000 inhabitants. Secondary clusters were located in the Southeast and Northeast regions (Figure 2, panel B; Table).

**Table:** Significant spatiotemporal clusters of schistosomiasis-related deaths as defined by space-time scan statistic, by municipality of residence, Brazil, 2000–2011*

Cluster†	Period	No. munis.	States	Region(s)	Radius, km	Death rate‡	No. observed/no. expected	LLR	RR	p value
1	2001–2006	191	Paraiba, Pernambuco, Alagoas	Northeast	179.3	4.0	2,150/214.6	3,257.52	12.96	<0.001
2	2006–2011	996	Sergipe, Bahia, Goiás, Minas Gerais, Espírito Santo, Rio de Janeiro	Northeast, Central-West, Southeast	688.8	0.6	1,161/734.2	116.79	1.69	<0.001
3	2000–2005	27	São Paulo	Southeast	38.7	0.5	572/427.9	23.16	1.36	<0.001

*Space-time scan statistic is described in the online technical appendix (http://wwwncd.cdc.gov/EID/article/21/10/14-1438-Techapp1.pdf). LLR, log- likelihood ratio test; munis, municipalities; RR, relative risk for the cluster compared with the rest of the country.  †The most likely or primary clusters (1) and secondary clusters (2 and 3) were detected by the LLR. The most likely cluster was defined as the one with the maximum LLR. ‡Average annual rate of death for schistosomiasis per 100,000 inhabitants during the cluster period.

## Conclusions

In this nationwide population-based study in Brazil, we found a heterogeneous geographic pattern of schistosomiasis-related deaths. Independently from the spatial statistical approach, high-risk clusters for schistosomiasis-related deaths were identified mainly in the highly schistosomiasis-endemic areas along the east coast of the Northeast Region, particularly in the states of Alagoas, Pernambuco, Sergipe, and Bahia and extending north of Minas Gerais and Espírito Santo States in the Southeast (*4**,**9**,**10*). These areas have ecologic and geographic conditions favorable to schistosomiasis: presence and proliferation of the intermediate snail host, poor living conditions, and inadequate sanitation (*10*). Reducing severe forms of schistosomiasis will require controlling transmission by implementing measures such as promoting basic sanitation and health education (*4**,**11*).

We also identified high rates of schistosomiasis-related deaths in areas where the disease is not endemic and has no focal transmission. The continuing emergence of schistosomiasis, characterized by the appearance of new foci in nonendemic areas and by urbanization of the disease, may be related to internal migration, increasing urban agglomeration, wide distribution of intermediate hosts, and discontinuation of disease control measures (*9*). High levels of internal migratory movement, the spread of snail intermediate hosts, and poor sanitary conditions increase the risk for establishing new foci in Brazil (*9**,**12*). For example, Rondônia state in North Brazil recorded increasing numbers of confirmed cases in recent years (*13*). Most cases and deaths in this state were not autochthonous but were identified in migrants coming from schistosomiasis-endemic regions of Brazil (*13*). The presence of potential intermediate hosts has been confirmed in Rondônia, increasing the possibility that the disease will establish there (*12**,**13*). In other regions of the world, transmission seems to establish in non–disease-endemic areas; on the island of Corsica (France), several tourists have been infected with *Schistosoma haematobium* while bathing in local rivers (*14*).

Although schistosomiasis is a disease typical of poor rural areas, intensified urbanization in recent decades has led to increasing numbers of urban cases and deaths (*11**,**15*). Municipalities that recorded the highest number of deaths were concentrated in Brazilian state capitals, especially in São Paulo (São Paulo State), Recife (Pernambuco State), Maceió (Alagoas State), and Belo Horizonte (Minas Gerais State). Most cases probably originated with persons coming from schistosomiasis-endemic rural areas and migrating to capital cities and metropolitan regions in search of improved living conditions and increased access to specialized health services (*11*).

Furthermore, development and management of water resources projects can introduce schistosomiasis into areas not previously endemic for the disease (*2*). The transposition of the largest river in the Northeast Region (São Francisco River), set to begin in 2016, may contribute to disease outbreaks through dispersion of intermediate hosts to areas not previously schistosomiasis endemic and through increased migratory activities of construction workers and their families (*4*).

Our study is subject to limitations. Because we used secondary death data, deaths may be underreported (*4*), despite progress achieved in registration of deaths (estimated proportion of deaths reported increased from 91.0% in 2000 to 94.2% in 2011; http://tabnet.datasus.gov.br/cgi/idb2012/a1801b.htm). Furthermore, schistosomiasis as an underlying cause of death may be underreported because it could be coded as a complication or illness associated with schistosomiasis (e.g., gastrointestinal bleeding, portal hypertension, esophageal varices) (*4**,**11*). To reduce this error, we collected information from data showing multiple causes of death (underlying and associated causes) and identified all death certificates that mentioned schistosomiasis. In addition, identifying areas of high transmission of disease by using death data must be approached with care. Schistosomiasis is a chronic disease, and death may result from an infection acquired many years earlier (*4*). Because of geographic migration of infected persons, place of residence at time of death may not be the place where the infection was acquired (*5*). Another limitation is the uncertainty of population estimates during intercensus years used in calculations of rates, especially estimates for years far from census years (2000 and 2010).

Our results indicate spatiotemporal heterogeneity of schistosomiasis-related deaths in Brazil over a 12-year period. High-risk clusters were located mainly in highly schistosomiasis-endemic areas. Disease control programs should increase geographic coverage, intensify and focus efforts to reduce transmission and prevent severe illnesses and deaths, and prevent establishment of schistosomiasis in areas where it is not yet endemic.

Technical Appendix Detailed description of methods used in statistical analyses for study of schistosomiasis-related deaths in Brazil, 2000–2011.
