# Supplementary material for: Spatiotemporal Patterns of Schistosomiasis-Related Deaths, Brazil, 2000–2011
Source: Emerg Infect Dis. 2015 Oct;21(10):1820–3. doi: 10.3201/eid2110.141438 (PMC4593422; doi:10.3201/eid2110.141438)
Supplement: Technical Appendix — Detailed description of methods used in statistical analyses for study of schistosomiasis-related deaths in Brazil, 2000–2011. [file 14-1438-Techapp-s1.pdf]

Article DOI: http://dx.doi.org/10.3201/eid2110.141438

Spatiotemporal Patterns of Schistosomiasis-Related Deaths, Brazil, 2000–2011

Technical Appendix

Detailed Description of Statistical Analyses

Crude Death Rates

We used schistosomiasis-related death data for all 5,565 Brazilian municipalities of residence (territorial division as of 2010) as geographic units of analysis. We excluded deaths with unknown municipality of residence. We calculated average annual crude (unsmoothed) death rates at the municipality level over the study period (2000–2011). Average death rates were calculated by dividing the average annual number of schistosomiasis-related deaths by the population size in the middle of the study period, expressed per 100,000 inhabitants. Indicators were calculated by using Stata software version 11.2 (Stata Corp LP, College Station, TX, USA); mapping was performed by using ArcGIS software version 9.3 (Esri, Redlands, CA, USA).

Smoothed Death Rates

We calculated smoothed death rates (per 100,000 inhabitants) by using the Local Empirical Bayes method. This approach aims to reduce random variations and provides greater stability of death rates in municipalities with small populations and rare events; it also reduces possible variation resulting from underreporting of deaths due to operational factors (*1*). Death rates were adjusted by incorporating data from neighboring spatial units (municipalities) with contiguous boundaries (*1*). Empirical Bayes smoothing estimates of schistosomiasis-related death rates were determined by using the "*Bayes Empírico Local*" approach of the TerraView software version 4.2 (Instituto Nacional de Pesquisas Espaciais São Paulo, Brazil; http://www.dpi.inpe.br/terraview_eng/index.php). Data were transferred for mapping with the ArcGIS software version 9.3 (Esri).

Spatial Cluster Analysis

We evaluated the presence of global spatial autocorrelation by using Global Moran's I statistic (*2*). Moran's I index ranges from –1 to +1: values close to zero indicate spatial randomness; positive values indicate positive spatial autocorrelation; and negative values indicate negative spatial autocorrelation (*2*). Spatial autocorrelation was considered significant if the p value was <0.05. We evaluated the existence of local autocorrelation (Local Index of Spatial Association [LISA]) by using Local Moran's I statistic (*3*). LISA was used to identify significant hot spots (High-High: high-values spatial clusters), cold spots (Low-Low: low values spatial-clusters), and spatial outliers (High-Low: high values surrounded with low values or Low-High: Low values surrounded with high values) of schistosomiasis-related death rates (*3*). For spatial representation of the Local Moran's index, Moran maps were used that considered municipalities with statistically significant differences (p<0.05). Spatial analysis was performed by using the Spatial Analyst extension of the ArcGIS software version 9.3 (Esri).

Scan Space-Time Cluster Analysis

We used retrospective Kulldorff's space-time scan statistics to identify high-risk spatiotemporal clusters for schistosomiasis-related deaths (*4*,*5*). The test is performed by gradually scanning a window across time and space and noting the number of expected and observed deaths at each municipality. This window is defined as a cylinder in which the circular geographic base corresponds to space and height corresponds to years under consideration (*5*). For scanning windows, we used the following parameters: Poisson probability model, time aggregation of 1 year, a maximum spatial cluster size of 30% of the population at risk, and a maximum temporal cluster size of 50% of the study period. For each circle, the log likelihood ratio (LLR) test is computed on the basis of the number of observed and expected cases within the window, compared with the ratio outside the window (*5*). We computed statistical significance of detected clusters by using 999 Monte Carlo replications. The window with the maximum LLR was defined as the most likely cluster or primary cluster (least likely to have occurred by chance) (*5*). We reported statistically significant clusters with an indicated p value <0.05. Scan space-time analysis was performed by using SaTScan software version 9.1.1 (Harvard Medical School, Boston, MA, USA; Information Management Service Inc., Silver Spring, MD, USA; www.satscan.org/), and mapping was done by using the ArcGIS software version 9.3 (Esri). The SaTScan program was used to

obtain reported observed cases, expected cases, relative risk, annual deaths per 100,000 inhabitants, and locations of specific cluster.

References

1. Assunção RM, Schmertmann CP, Potter JE, Cavenaghi SM. Empirical Bayes estimation of demographic schedules for small areas. Demography. 2005;42:537–58. PubMed http://dx.doi.org/10.1353/dem.2005.0022

2. Cliff AD, Ord JK. Spatial processes: models & applications: London: Pion. 1981.</bok>

3. Anselin L. Local indicators of spatial association–LISA. geographical analysis. 1995;27:93–115.

4. Kulldorff M. A spatial scan statistic. Comm Stat Theory Methods. 1997;26:1481–96. http://dx.doi.org/10.1080/03610929708831995

5. Kulldorff M. SaTScan user guide for version 9.0. 2010 [cited 2014 Jul 15]. http://www.satscan.org/
